# Post COVID-19 syndrome in a prospective cohort study of Egyptian patients

**DOI:** 10.1186/s43166-021-00104-y

**Published:** 2022-02-02

**Authors:** Dalia Mohamed Gamal, Rehab Ali Ibrahim, Sara Farid Samaan

**Affiliations:** 1 Department of Internal Medicine, Faculty of Medicine, Ain-Shams University, Cairo, Egypt; 2 Department of Physical Medicine, Rheumatology and Rehabilitation, Faculty of Medicine, Ain-Shams University, Cairo, Egypt

**Keywords:** Coronavirus, COVID-19, PCFS scale, Post COVID-19 syndrome

## Abstract

**Background:**

Post-coronavirus disease (COVID-19) syndrome is defined as the persistence of symptoms for more than 3 to 12 weeks after infection with the COVID-19 virus that cannot be attributed to another etiology. This study was conducted in our university hospital aiming to analyze the medium-term persistent symptoms in post-COVID-19 patients through a comprehensive and structured clinical assessment and evaluating the incidence, association, and risk factors of the post COVID-19 symptoms and their effect on the functional status of the survivors.

**Results:**

Of 170 recruited individuals, about 66 (38.82%) reported post-COVID-19 symptoms. Post-viral fatigue was the most common symptom (23.5%), followed by arthralgia and myalgia in 32 patients (18.8%). Lower functional status was reported among some of the survivors which can be attributed to the severity of the disease and the presence of post-COVID symptoms among these patients. The post-COVID-19 syndrome showed an association with patient age, severity of the disease, and the presence of preexisting comorbidities.

**Conclusion:**

A significant functional impact was found in some COVID-19 survivors after COVID-19 infection. Age, severity of the disease, and presence of preexisting comorbidities are critical risk factors for the development of post-COVID-19 syndrome.

## Background

The narrative of COVID-19 began in December 2019 in Wuhan, China, with a cluster of pneumonia patients of unclear etiology [1]. It quickly spread as an outbreak there and the National Health Commission of the Republic of China later stated that the outbreak was caused by a novel coronavirus now known as COVID-19. The virus was declared a pandemic by World Health Organization (WHO) on March 11, 2020 [2]. The disease has begun to spread around the world including Egypt. The first case was recorded in Egypt on February 14, 2020 [3]. Since then, Egypt has shown a gently rising trajectory up till now [4].

Acute COVID-19 patients exhibited a wide range of symptoms ranging from minor respiratory symptoms to a severe type of pneumonia requiring mechanical ventilation and finally progressing to acute respiratory distress syndrome or multi-organ failure. Unfortunately, the fight against COVID-19 does not appear to end with the detection and treatment of acute disease. Studies have revealed a high incidence of persistent symptoms after acute infection in 40 to 90% of patients resulting in the term “long COVID-19” or “post COVID-19 syndrome” [5].

Because COVID-19 is a new virus, there is currently no agreement on the definition of post-COVID-19 syndrome. According to the National Institute for Health and Care Excellence (NICE), the Scottish Intercollegiate Guidelines Network (SIGN), and the Royal College of General Practitioners (RCGP), post COVID-19 was defined as “Signs and symptoms that arise during or following an infection compatible with COVID-19 that persist for more than 12 weeks and are not explained by an alternative diagnosis” [6]. Greenhalgh et al. defined post-COVID syndrome as COVID-19-related disease that lasts longer than 3 weeks after the beginning of symptoms and chronic COVID-19 as symptoms that last longer than 12 weeks after the onset of symptoms [7]. According to Amenta et al., for those who stay in the hospital at 3 weeks following symptom onset the post-acute phase begins when the patient is discharged from inpatient acute therapy [8]. Recently, the University of Cincinnati Medical Center’s recommended criteria for COVID-19 sequelae; they divided long COVID-19 syndrome into five categories based on the initial symptoms, onset time, length of symptoms, and period of quiescence [9].

People with post-COVID-19 syndrome continued to experience one or more symptom such as generalized discomfort, exhaustion, persistently high temperature, and psychological issues. These symptoms do not just impact people who have had a bad case of the disease but can also affect people with mild course of the disease. Since the data of people experiencing long COVID-19 symptoms is still unclear [10], this study was conducted in our university hospital aiming to analyze the medium-term persistent symptoms in post COVID-19 patients through a comprehensive and structured clinical assessment and evaluating the incidence, association, risk factors of the post COVID-19 symptoms, and their effect on the functional status of the survivors.

## Methods

This single-center prospective cohort study was carried out at COVID-19 unit of Ain Shams  university hospitals.

### Participants

Patients with COVID-19 who presented to Ain Shams University hospitals triage and inpatient departments were screened for the study. Patients over the age of 18 with confirmed severe acute respiratory syndrome coronavirus 2 (SARS-CoV-2) positivity on reverse transcription polymerase chain reaction (RT-PCR) were eligible to participate. COVID-19 instances that were asymptomatic or critical, as well as individuals who refused to participate, were omitted from the study. All patients gave their informed written consent, after clinical recovery and viral clearance by two negative RT-PCR 48 h apart, the patients were monitored for at least one and a half month.

### Study design

Patients’ demographics, clinical signs and symptoms, comorbidities, and routine tests, such as complete blood count, C-reactive protein, creatinine, random blood sugar, alanine aminotransferase, and D-dimer, and high-resolution computed tomography chest (HRCT), were all suggested on enrolment. Patients were classified into 3 groups: mild, moderate, and severe. Mild disease was defined as symptoms of an upper respiratory tract viral infection, including mild fever, cough (dry), sore throat, nasal congestion, malaise, headache, muscle pain, anosmia, or malaise, according to WHO guidelines. Moderate disease was defined as respiratory symptoms such as cough and shortness of breath in the absence of indicators of severe pneumonia. Severe illness was defined as severe dyspnea and tachypnea (breathing rate of more than 30 breaths per minute). Clinical improvement was defined as normal body temperature for at least 3 days, significant improvement in respiratory symptoms (respiratory rate 25 breaths per minute and no dyspnea), oxygen saturation (SpO2) > 93% with no oxygen inhalation assistance, and no hospitalization for any pathology or clinician assessment [11]. All the patients were followed up in our post-COVID-19 clinic after one and half month for having persistent symptoms like fatigue, fever, dyspnea, and arthralgia, in addition to functional status assessment after COVID-19 infection using the Post COVID-19 Functional Status scale (PCFS) [12].

### Statistical analysis

All data was gathered, tallied, and entered into IBM SPSS version 23 (released in 2015). Version 23.0 of IBM SPSS Statistics for Windows (Armonk, NY: IBM Corp.). Quantitative data with parametric distribution was displayed as mean, standard deviation, and ranges, but non-parametric data was displayed as median with inter-quartile range (IQR). Numbers and percentages were also used to represent qualitative characteristics. The chi-square test was used to compare the qualitative data of the two groups. The independent *t* test was used to compare two independent groups with quantitative data and parametric distribution, while the Mann-Whitney test was used to compare two independent groups with non-parametric distribution. Univariate and multivariate logistic regression analysis was used to predict the risk factors for the occurrences of post COVID-19 symptoms and factors that affects the functional status with its odds ratio (OR) and 95% confidence interval (CI). The confidence interval was set to 95% and the margin of error accepted was set to 5%. So, the *p* value was considered significant at the level of *P* ≤ 0.05.

### Ethical consideration

Approval of the study conduction was obtained from the Ethical Committee. Written informed consent was obtained from all participants.

## Results

This cohort Egyptian study was conducted on 170 patients, the median age of the participants was 57 years, there were 64 male patients and 106 female patients with male to female ratio 1:1.65, most of the patients had 1 or more comorbid diseases where 67 (39.4%) patients were diabetic, 73 (42.9%) were hypertensive, 25 (14.7%) patients with ischemic heart disease (IHD), 5 (2.9%) patients with cancer, 11 (6.5%) patients with autoimmune diseases (AID), 12 (7.1%) patients with stroke, 7(4.1%) patients with chronic renal failure (CRF), 8 (4.7%) patients with chronic liver disease (CLD), and 9 (5.3%) patients with chronic obstructed lung disease COPD (COPD) (Table 1).

**Table 1:** Descriptive of demographic data, clinical characteristic, and severity of COVID-19 among our patients

Parameter measured	No.	%
Age		
Mean ± SD, 55.46 ± 16.07		
Median, 57 (45‑67)		
Range, (18‑94)		
Sex		
Male	64	37.6%
Female	106	62.4%
Comorbidities		
DM	67	39.4%
HTN	73	42.9%
IHD	25	14.7%
Cancer	5	2.9%
AID	11	6.5%
STROKE	12	7.1%
CRF	7	4.1%
CLD	8	4.7%
COPD	9	5.3%
COVID-19 symptoms		
Fatigue	153	90.0%
Fever	118	69.4%
Dyspnea	117	68.8%
Arthralgia	127	74.7%
Myalgia	127	74.7%
Cough	122	71.8%
Anosmia	27	15.9%
Rhinitis	13	7.6%
Headache	55	32.4%
Chest tightness	70	41.2%
Sore throat	26	15.3%
Nausea	20	11.8%
Diarrhea	18	10.6%
Loss of taste	12	7.1%
COVID-19 severity		
Mild	95	55.9%
Moderate	47	27.6%
Severe	28	16.5%

*SD* standard deviation, *DM* diabetes mellitus, *HTN* hypertension, *IHD* ischemic heart disease, *AID* autoimmune disease, *CLD* chronic liver disease, *CRF* chronic renal failure, *COPD* chronic obstructive lung disease

At the time of initial presentation of the patients, 153 patients were presented with fatigue, 118 patients with fever, 117 patients with dyspnea, 127 patients with arthralgia and myalgia mainly in the form of low back pain, 122 patients with dry cough, 27 patients with anosmia, 13 patients with rhinitis, 55 patients with headache, 70 patients with chest tightness, 26 patients with sore throat, 20 patients with nausea, 18 patients with diarrhea, 12 patients with loss of taste, and finally 119 patients were diagnosed to have pneumonia with CT chest (Table 1).

As regard severity of the disease, 95 (55.9%) patients were presented with mild disease, 47 (27.6%) patients with moderate disease, and 28 (16.5%) patients with severe disease according to WHO classification (Table 1).

### Post COVID-19 symptoms

Among the 170 recruited patients, COVID-19 symptoms persisted beyond recovery for one and half months in about 66 (38.82%). Post viral fatigue was the most prevalent persistent feature in about 40 patients, followed by arthralgia and myalgia in 32 patients in addition to other persistent symptoms as shown in Table 2.

**Table 2:** Descriptive of post COVID-19 clinical characteristics

Post COVID-19 symptoms	No.	%
Fatigue	40	23.5%
Fever	4	2.4%
Dyspnea	23	13.5%
Arthralgia	32	18.8%
Myalgia	32	18.8%
Cough	8	4.7%
Anosmia	2	1.2%
Rhinitis	2	1.2%
Headache	9	5.3%
Chest tightness	17	10.0%
Sore throat	0	0.0%
Nausea	2	1.2%
Diarrhea	1	0.6%
Loss of taste	1	0.6%

To identify risk factors for the occurrence of post COVID-19 symptoms, we obtained the odds ratios (OR) after conducting the logistic regression analysis. The univariate analysis showed that increase age with *P* < 0.001, presence of comorbidities (OR, 2.44; 95% CI, 1.2‑4.9; *P* = 0.012) were significantly associated with increased incidence of presence of post COVID-19 symptoms (Table 3).

**Table 3:** Logistic regression analysis for prediction of risk factors for post COVID-19 symptoms

	Post COVID-19 symptoms		OR (95 CI%)	Test of significance		
	No	Yes				
	Mean ± SD, ***N*** (row %)	Mean ± SD, ***N*** (row %)		Value	***p*** value	Sig.
Age	51.54 ± 16.35	61.97 ± 13.35		***t*** = −4.527	< 0.001	S
Sex						
Male	39 (60.94%)	25 (39.06%)	0.908 (0.48‑1.7)	***X***^***2***^ ***=*** 0.088	0.767	NS
Female	67 (63.21%)	39 (36.79%)				
Co-morbidities						
No	43 (75.44%)	14 (24.56%)	2.44 (1.2‑4.9)	***X***^***2***^ ***=*** 6.255	0.012	S
Yes	63 (55.75%)	50 (44.25%)				

*OR* odd ratio, *CI* confidence interval, *t* student *t* test value, *X*^2^ chi square test value, statistical significance at *p* ≤ 0.05

### Post COVID-19 functional status

Some patients reported reduction in function post COVID-19, in our study, functional status was assessed using the PCFS scale where 52 patients were found to be grade 1 (negligible limitation in everyday life, persistent symptoms, pain, depression, or anxiety), 41 patients with grade 2 (slight functional limitations), 15 patients with grade 3 (moderate functional limitation), and 2 patients with grade 4 (severe functional limitation) (Table 4).

**Table 4:** Descriptive of post COVID-19 functional status scale in our patients after one and half months

Parameter measured	Mean/***N***	SD %	Median (IQR)	Range
PCFS scale grade (after 1.5 months)				
0	60	35.3%		
1	52	30.6%		
2	41	24.1%		
3	15	8.8%		
4	2	1.2%		
PCFS scale grade (after 1.5 months)	1.10	1.02	1 (0‑2)	(0‑4)

*PFCS* Post COVID-19 Functional Status Scale, *IQR* interquartile range, *SD* standard deviation

To identify risk factors that may affect functional status, we obtained the odds ratios (OR) after conducting the logistic regression analysis. The univariate analysis showed that increase age with *P* < 0.001, presence of comorbidities (OR, 8.273; 95% CI, 4.0‑17.0; *P* < 0.001) were significantly associated with poor functional status in patients with post COVID-19 symptoms (Table 5).

**Table 5:** Logistic regression analysis for prediction of risk factors that affect functional status in patients with post COVID-19 symptoms

	PCFS (1.5 Months)		OR (95 CI%)	Test of significance		
	No	Yes				
	Mean ± SD, ***N*** (row %)	Mean ± SD, ***N*** (row %)		Value	***p*** value	Sig.
Age	42.68 ± 14.66	62.44 ± 12.05		***t =*** −8.923	< 0.001	S
Sex						
Male	24 (37.5%)	40 (62.5%)	1.167 (0.61‑2.2)	***X***^***2***^ ***=*** 0.219	0.64	NS
Female	36 (33.96%)	70 (66.04%)				
Co-morbidities						
No	38 (66.67%)	19 (33.33%)	8.273 (4.0‑17.0)	***X***^***2***^***=*** 36.957	<0.001	S
Yes	22 (19.47%)	91 (80.53%)				

*OR* odd ratio, *CI* confidence interval, *t* student *t* test value, *X*^2^ chi-square test value; statistical significance at *p* ≤ 0.05

The functional status post COVID-19 was found to be affected by severity of the disease and with the presence of post COVID-19 symptoms with *p* value < 0.001 (Table 6).

**Table 6:** Relation between functional status with disease severity and the presence of post COVID-19 symptoms

	PCFS post (1.5 months)		Chi-square test		
	No	Yes			
	***N*** (row %)	***N*** (row %)	***X***^***2***^	***p*** value	Sig.
Severity					
Mild	59 (62.1%)	36 (37.9%)	67.814	< 0.001	S
Moderate	1 (2.1%)	46 (97.9%)			
Severe	0 (0.0%)	28 (100.0%)			
Post COVID-19 symptoms					
No	50 (83.33%)	56 (50.91%)	17.388	< 0.001	S
Yes	10 (16.67%)	54 (49.09%)			

*X*^2^ chi square test value, statistical significance at *p* ≤ 0.05

## Discussion

This study included 170 Egyptian patients diagnosed with acute COVID-19 syndrome recruited from Ain Shams University hospitals. We attempted to include patients with mild (59.5%), moderate (27.6%), and severe disease (16.5%). The proportion of women with COVID-19 in our study was higher than that of men and the mean age of the patients was 55 years. Most of the patients initially presented with fatigue, followed by other symptoms such as fever, dyspnea, arthralgia, and myalgia as reported in earlier research [13].

According to Becker et al. [9] who classified COVID-19 sequelae into five categories, type 2 was characterized by symptoms persisting 6 weeks from the onset of illness. So, after a one-and-a-half-month follow-up, we discovered that about 38.8% of the patients still had mild COVID-19 symptoms, which was consistent with many studies including one from the USA, which found that 35% of patients did not return to their previous health status even after 3 weeks of COVID-19 positivity [14]. Another prospective study by Carvalho-Schneider et al., done on 150 mild and moderate COVID-19 patients showed the presence of dyspnea and asthenia, respectively, in 30 and 40% of patients at 2 months of follow-up [15], which is partially consistent with our results where dyspnea and fatigue were persistent in 13.5% and 23.5% of our patients respectively. Another large study on 355 mild-to-severe COVID-19 patients reported that about 46% of patients had persistent post COVID-19 symptoms at 1 month of follow-up that were more evident in the female patient with fatigue being the main prevalent symptom going with the results of our study [16]. On the other side, an Italian study, Carf et al. [17] followed-up the patients with mild to severe disease who met the WHO criteria for discontinuation of quarantine (no fever for consecutive 3 days, improvement in other symptoms, and two negative test results for SARS-CoV-2, 24 h apart) and found that approximately 87% of the cases had persistence of at least one symptom, where the most common symptoms in the form of fatigue, dyspnea, joint, and chest pain respectively in 53.1%, 43.4%, 27.3%, and 21.7% of patients at 2 months of follow-up, their results seem to be in line with ours where fatigue, arthralgia, dyspnea, and chest tightness were the most common persistent long-term symptoms.

Various studies have identified a variety of post-COVID-19 symptoms. According to our findings both patients with mild and severe instances of COVID-19 can acquire post-COVID-19 symptoms. The most common post-COVID-19 symptom, following recovery according to most research, is chronic fatigue which is followed by cough, dyspnea, and widespread musculoskeletal pain [18]. Based on a recent systematic review by Salamanna et al., chronic fatigue is clearly considered the most common long-term symptom in mild-to-severe COVID-19 patients [19]. Fatigue, post-exercise dyspnea, and persistent musculoskeletal discomfort were found in 23.5%, 13.5%, and 18.8% of the participants in our study respectively. The cause of fatigue predominance was mostly unknown, several investigations have shown that immune system changes caused by viral infections may be the cause of weariness by triggering an inflammatory immune response [19, 20]. Therefore, it might be necessary to examine cytokine networks in COVID-19 survivors to see if the “cytokine storm” that occurred during the disease remains and contributes to these long-term problems. Several studies have demonstrated persistent HRCT lung abnormalities after 60 days from the initial presentation. A decreased diffusion capacity due to loss of lung volume is the most commonly reported pathophysiologic impairment in post-acute effects of COVID-19 which is directly related to the severity of acute illness [21].

Some of prior studies failed to identify risk factors for post-COVID-19 syndrome, according to the current study even patients with modest symptoms at the time of presentation can acquire post-acute COVID-19 symptoms, there was a strong association between post-COVID-19 syndrome and age of the patients, existence of underlying comorbidities and severity of the condition.

Functional status among COVID-19 survivors exhibited affection may be due to persistence of post COVID-19 symptoms. In our study, we used the PCFS scale to assess functional status among COVID-19 survivors one and a half month after initial infection. We found that 52 patients were grade 1 (negligible limitation), 41 patients were grade 2 (slight limitation), 15 patients were grade 3 (moderate limitation), and 2 patients were grade 4 (severe limitation).

Lower functional status was found to have a statistically significant relationship with patient age, disease severity, presence of post COVID-19 symptoms, and finally the presence of preexisting comorbidities. This was partially in accordance with the results of Hussein et al., who found significant association of PCFS with age and presence of comorbidities [22]. Our study findings revealed that respondents with chronic medical conditions (HTN, DM, IHD, cancer, arthritis) reported significantly lower functional status scores. Our findings are in line with those of a Moroccan study, which found that the COVID-19 pandemic had a mostly negative impact on QOL and well-being of those with chronic medical conditions [23]. Another study also supported the vulnerability of individuals with chronic medical problems to experience poor quality of life during the first wave of COVID-19, which explains the finding that “chronic illness or a self-evaluation of poor health is associated with increased psychological distress” [24].

This study has strengths as being one of the few studies done to assess functional status and long-term dysfunction among post COVID-19 survivors using a validated, specific, and an easy tool that can be easily applied to the patients which is the PCFS. In addition, our study identifies the most prevalent long-term symptoms which might impede return to pre-COVID-19 infection functional capacity. Our study has some limitations. First, it is a single center study. Second, the follow-up period for patients was limited (1 and half months after disease onset) and third, the small sample size. Further longitudinal studies are recommended to assess the long-term effect of COVID-19 infection and the association between the persistence of long-term symptoms and proinflammatory cytokines.

## Conclusions

We found that some COVID-19 survivors reported a significant impact on their functional status after the virus. Age, disease severity, and the existence of pre-existing comorbidities are all important risk factors for developing post-COVID-19 syndrome.

## Data Availability

All data generalized and/or analyzed during the current study are available with principal author upon reasonable request.

## Abbreviations

AID: Autoimmune diseases
CLD: Chronic liver disease
COPD: Chronic obstructed pulmonary disease
COVID: Coronavirus disease
CRF: Chronic renal failure
DM: Diabetes mellitus
HRQHRCT: High-resolution computed tomography
HTN: Hypertension
IHD: Ischemic heart disease
PCFS: Post COVID-19 Functional Status Scale
RT_PCR: Reverse transcriptase polymerase chain reaction
SARS: Severe acute respiratory syndrome
WHO: World Health Organization
